# Managing Essential Tremor

**DOI:** 10.1007/s13311-020-00899-2

**Published:** 2020-09-11

**Authors:** Franziska Hopfner, Günther Deuschl

**Affiliations:** 1 Department of Neurology, UKSH, Christian-Albrechts-University Kiel, Rosalind-Fraenklinstr. 10, 24105 Kiel, Germany; 2 Department of Neurology, Hannover Medical School, Hannover, Germany

**Keywords:** Tremor, essential tremor, managing of essential tremor, treatment of essential tremor, deep brain stimulation, focused ultrasound

## Abstract

Essential tremor is one of the most common tremor syndromes. According to the recent tremor classification, tremor as a symptom is defined as an involuntary, rhythmic, oscillatory movement of a body part and is classified along two axes: axis 1—defining syndromes based on the clinical features such as historical features, tremor characteristics, associated signs, and laboratory tests; and axis 2—classifying the etiology (Bhatia et al., Mov Disord 33:75–87, 2018). The management of this condition has two major approaches. The first is to exclude treatable etiologies, as particularly during the onset of this condition the presentation of a variety of etiologies can be with monosymptomatic tremor. Once the few etiologies with causal treatments are excluded, all further treatment is symptomatic. Shared decision-making with enabling the patient to knowledgeably choose treatment options is needed to customize the management. Mild to moderate tremor severity can sometimes be controlled with occupational treatment, speech therapy of psychotherapy, or adaptation of coping strategy. First-line pharmacological treatments include symptomatic treatment with propranolol, primidone, and topiramate. Botulinum toxin is for selected cases. Invasive treatments for essential tremor should be considered for severe tremors. They are generally accepted as the most powerful interventions and provide not only improvement of tremor but also a significant improvement of life quality. The current standard is deep brain stimulation (DBS) of the thalamic and subthalamic region. Focused ultrasound thalamotomy is a new therapy attracting increasing interest. Radiofrequency lesioning is only rarely done if DBS or focused ultrasound is not possible. Radiosurgery is not well established. We present our treatment algorithm.

**Electronic supplementary material:**

The online version of this article (10.1007/s13311-020-00899-2) contains supplementary material, which is available to authorized users.

## Introduction

The management of essential tremor covers two important aspects [1]. The first is diagnostic with the main questions is for accuracy of diagnosis and not to overlook specifically treatable etiologies. A new tremor classification has laid new foundations for this [2]. The second is to understand and interpret the needs of the patient. The spectrum of complaints varies considerably from patient to patient. Essential tremor (ET) is a lifelong lasting syndrome and young patients may have different demands than the elderly. Essential tremor is a progressive syndrome and patients early during the course of the disease may have different problems from those with advanced ET. Men and female can both have essential tremor but the complaints may differ for them. For all these aspects, it is important to know about the spectrum of manifestations of the condition but also about the ways to measure tremor severity and complaints. The selection of treatment is critically depending on the knowledge of these subjective complaints.

## The Essential Tremor Syndrome and Its Etiologies

The new classification of MDS is based on a 2-axis principle. The clinical phenomenology of tremor is mapped on axis 1 (clinical description) and the tremor etiology on axis 2. The axis 1 descriptors (historical features, tremor characteristics, associated signs, and additional laboratory tests) allow a syndromic classification based on these features. Essential tremor is defined as an isolated tremor syndrome of bilateral upper limb action tremor with or without tremor in other locations of at least a 3-year duration. Other neurologic symptoms sufficient to make an additional neurologic syndrome diagnosis (e.g., Parkinson syndrome, dystonia, cerebellar disease, peripheral neuropathy…) must be absent. Based on clinical experience, such patients very often have slight symptoms called “soft signs” (impaired tandem gait, obvious tremor at rest, questionable dystonic posturing, memory impairment etc.) which are suspicious but not sufficient to allow a second diagnosis. These patients are defined as having “essential tremor plus (ET+).” This entity has been defined because an unknown number of these patients may later develop other diagnoses and may be a matter of discussion even among specialists [3]. On the long run, closer definitions are needed here. Compared to the previous definition of essential tremor in the 1998 classification [4], two recent studies have shown that only ~ 15% [5] or 46% [6] are classified as ET but more than 80% [5] or 54% [6] were classified as ET+ according to the 2018 classification [2]. The message is that the majority of ET patients seems to have such soft signs.

If a patient is diagnosed with an ET− or ET+ syndrome, the next question is for a possible etiology. Etiologies might be hyperthyroidism, hypoglycemia, and medication-induced tremors which have to be excluded with careful medical history and lab tests. Table 1 summarizes the etiologies which can on rare occasions be associated with tremor and can be rarely confused with ET. Certainly, the majority of them will only be addressed with further diagnostics if additional symptoms suggest one of these conditions. Most of the patients with the syndromes of ET or ET+, however, will have no identifiable cause [7]. Certainly, causal treatments will be recommended if a treatable etiology is found.

**Table 1:** List of different tremor entities which can present with an ET− or ET+ syndrome. Untreatable causes include monogenetic causes, and chromosomal aberrations. Much of the following diseases are causally treatable: infectious and inflammatory diseases, autoimmune neuropathies and spinal muscular atrophies, paraneoplasias, endocrine or metabolic diseases, brain lesions of different etiologies, medications, toxins causing tremor, psychoactive drugs associated with tremor and affective changes, and consequences of physical exertion related with tremor

Possible etiologies which can present with an tremor syndrome	
Syndromes attributable to selected genes	Frontotemporal dementias, dystonia, Wilson’s disease, neuroferritinopathy, Lesch-Nyhan syndrome, pantothenate kinase–associated neurodegeneration (PEKAN), X-linked Parkinson-dystonia syndrome, primary familial brain calcification, Roussy-Lévy syndrome, hereditary neuropathies, spinocerebellar ataxia (types 12, 20, 27)
Syndromes attributable to selected chromosomal aberrations	Fragile X syndrome, Prader-Willi, ataxia telangiectasia, XYY syndrome, XXY (Klinefelter syndrome) XXYY syndrome, Angelman syndrome
Syndromes attributable to trinucleotide repeat diseases	Spinocerebellar ataxia (types 1, 2, 3, 6, 7, and 17), fragile X syndrome, dentatorubral-pallidoluysian atrophy
Parkinson syndromes	Parkinson’s disease, multiple system atrophy, corticobasal degeneration
Lysosomal storage disorders	Gaucher’s disease, Niemann-Pick disease, type C, action myoclonus-renal failure syndrome
Mitochondrial diseases	Syndrome of mitochondrial spinocerebellar ataxia and epilepsy (POLG-related disorders), Leigh syndrome, recessive monogenetic parkinsonian syndromes (DJ-1, PARKIN, PINK1)
Infectious and inflammatory diseases	Demyelinating diseases, encephalitis lethargica, subacute sclerosing panencephalitis, HIV, tuberculosis, syphilis, measles, typhoid, neuroborreliosis, bacterial or viral encephalitis, autoimmune encephalitis
(Autoimmune-) neuropathies and spinal muscular atrophies	Guillain-Barré syndrome, chronic inflammatory demyelinating polyneuropathy, Lewis-Sumner syndrome, gammopathy of undetermined significance (MGUS)
Paraneoplasia	Bronchial, breast, uterine, ovarian carcinoma with and without autoantibodies (Yo, Tr, VGKC, mGLuR1, Ri, Hu)
Endocrine or metabolic diseases	Liver and renal deficiency, hyperthyroidism, hyperparathyroidism, hypoglycemia
Brain lesions of different etiologies	brain tumors, craniocerebral trauma, electrical injuries, ischemia, bleeding, malformations
Drug-induced	Cytostatics (vincristine, cisplatin, paclitaxel, doxorubicin, cytosine-arabinoside, ifosfamide, 5-fluorouracil, methotrexate) Immunomodulators (ciclosporin, tacrolimus, interferons) Anticonvulsant drugs (valproate, carbamazepine, phenytoin, lamotrigine) Dopamine receptor blocker/medications depleting dopamine (neuroleptics, metoclopramide, tetrabenazine) Antidepressants (tricyclic antidepressants and selective serotonin/norepinephrine reuptake inhibitor, lithium) Antiarrhythmics (amiodarone, mexiletine, procainamide) Calcium antagonists (nifedipine, amlodipine) Hormones (thyroxine, calcitonin, progesterone, corticosteroids) Sympathomimetics (bronchodilators, β2-agonists) Phosphodiesterase inhibitors (theophylline, aminophylline caffeine)
Toxins	Mercury, lead, manganese, arsenic, cyanide, DDT, carbon monoxide, naphthalene, toluene, lindane
Psychoactive drugs	Caffeine, cocaine, nicotine, amphetamines, lysergic acid diethylamide, psilocybin and 3,4-methylenedioxymethamphetamine, alcohol withdrawal, withdrawal from benzodiazepines and others
Affective changes and consequences of physical exertion	Anxiety, excitement, stress, fatigue, physical exertion, cooling

In ET, gender influences the topographic distribution of tremor [8]. Female gender increases the likelihood of being (additionally to the hand tremor) affected from impairing head and/or voice tremor in ET [8, 9]. The association of hand tremor severity with midline tremor is stronger for males than for females [8].

## Differential Diagnostic Approaches for Tremor

Axis 1 includes tremor characteristics as the position most accentuating the tremor (rest, posture, action) which allows a syndromic approach for a precise classification (Table 2). The most common syndromes presenting with postural tremor are enhanced physiological tremor, essential tremor, and drug-induced tremor. The differential diagnosis must also include dystonic tremor and psychogenic tremor, while metabolic tremor caused by thyrotoxicosis should be considered in any recent-onset postural tremor.

**Table 2:** Combination of different activation conditions of tremor syndromes

Type of tremor	Rest	Posture	Action
Physiological		+ +	+
ET	−	+ +	+
ET plus	+	+ +	+
PD	+ +	+	+/−
Drug-induced	+	+ +	+
Endocrine/metabolic		+ +	+
Dystonic	+/−	+ +	+
Orthostatic		+ +	
Neuropathic		+ +	+
Holmes	+ +	+ +	+ +
Cerebellar		+/−	+ +
Psychogenic		+	+

+ +, typically present; +, may be present; +/−, occasionally present. *ET*: essential tremor; *PD*: Parkinson’s disease

Between essential tremor, dystonic tremor, and PD tremor syndromes, there is gray area in terms of diagnostic as resting and postural tremor may coexist. PD tremor most commonly occurs at rest, when the body part is relaxed and not in use, but can also be seen in the postural position, often referred to as re-emergent tremor [10]. A clinical test can distinguish PD tremor from ET tremor with a fairly high degree of reliability by assessing the suppression of rest tremor during movement [11].

Also distinguishing dystonic and essential tremor might be challenging in some patients. In the case of an accompanying vocal tremor, laryngoscopy accompanied by a speech specialist might be a useful diagnostic tool, to analyze tremorous vocalizations, to classify different tremors by vocal tremor patterns [12, 13].

For only few cases, electrophysiological or nuclear medicine methods must be applied going beyond purely clinical diagnostics. The dopamine transporter (DAT) is a presynaptic protein used as a biomarker for dopaminergic neurons. Single-photon emission tomography (SPECT) with cocaine derivative tracers binding to DAT can be used as a measure of dopamine deficiency. DAT-SPECT shows high selectivity to distinguishing PD and ET [14].

Certain tremor etiologies allow specific therapeutic approaches. Due to the knowledge of specific pathomechanisms which promote tremor-related syndromes, disease/tremor-modifying approaches are available to treat several tremor etiologies. Examples are listed in Table 3.

**Table 3:** Treatable conditions which may present mainly with tremor. Syndromes including tremor can be treated according to the underlying etiology of the disease.

Syndromes including tremor symptoms	Possible therapeutic interventions	Allocated example
Genetic syndromes involving metal metabolism	Chelators	Wilson’s disease
Storage disorders	Substrate reduction therapy, increase of lysosomal activity	Niemann-Pick type C
Syndromes attributable to trinucleotide repeat diseases	Antisense oligonucleotides	Spinocerebellar ataxia
Infection	Treatment of specific infection	Tick-borne encephalitis
Inflammation	Treatment of specific inflammation	Multiple sclerosis
Autoimmune neuropathies	Immunoglobulin therapy, corticosteroids	Chronic inflammatory demyelinating polyneuropathy
Paraneoplasia	Treatment of the underlying cause	Paraneoplastic anti-Yo cerebellar tremor ataxia syndrome
Endocrine or metabolic diseases	Treatment of the underlying cause	Hyperthyroidism
Medications	Change or omit the medication	Cyclosporine
Toxins	Remove toxin	Heavy-metal exposures
Psychoactive drugs	Omit substances	Caffeine
Affective changes and consequences of physical exertion	Relaxing techniques and thoughtful physical exertion/exercise	Progressive muscle relaxation

Syndromes attributable to monogenetic diseases or syndromes attributable to chromosomal aberrations are, with a few exceptions, not yet causally treatable.

If there are causal therapeutic approaches, i.e., if the etiology of the tremor is known (for example: side effect of a medication, concomitant symptom of another disease or syndrome, genetic cause, causal toxin), the tremor should be treated according to the needs of the condition and the individual suffering due to tremor.

## Overarching Aspects of the Treatment of Essential Tremor

If the etiology is unknown as for most of the patients only symptoms are treated, the threshold to treat should be based on patient-physician consensus. The decision on the extent for a symptomatic treatment should relate to the impairment subjectively felt by the patient due to both the motor symptoms and secondary impairment (psychosocial aspects). Not only objective tremor severity but also the patient subjective suffering and the related coping strategy are relevant. Important questions are the temporal development of tremor, facilitating and attenuating situations, topographic distribution of tremor, tremor components which are most disturbing, and the activities affected most by tremor. Complaints related to psychosocial aspects of the syndrome such as fear of stigmatization, social isolation, and depression due to tremor should impact the therapeutic decision-making. Some studies found an increased incidence of depression in ET [15, 16] while others found no differences [17] but this leaves the clinician with the need of an individual assessment of such symptoms for each patient. For tremors, the subjective perception of impairment is extremely variable between individuals and does not correlate very well with the measurable amplitude of the tremor, nor with the duration of the tremor and therefore the coping strategies differ [16]. Also, many patients, particularly those with mild symptoms, are mainly seeking advice on the etiology of the condition and want to have Parkinson’s disease excluded.

## How to Measure Success of Tremor Treatments

Tremor severity is measured with the Fahn/Tolosa/Marin rating scale (TRS) [18] or the Essential Tremor Rating Assessment Scale (TETRAS) rating scale [19] which both are clinimetrically well established but have known deficiencies [20]. The first has a clear ceiling effect for severe tremors, because the largest tremor amplitude for all phenomenological subtypes is > 4 cm, while the TETRAS has the most severe tremor amplitudes separated for the different extremities (hand tremor, > 20 cm; head tremor, > 5 cm; leg tremor, > 5 cm). On the other hand, TETRAS does not capture rest tremor. Both have subscales measuring tremor impairment (comparable to activities of daily living scales) and an objective assessment. Both are valid and reliable, sensitive to change and recommended [20]. There are scales measuring activities of daily living [21] but the performance parts of the TRS and the TETRAS may cover them. Quality of life is a broader concept and the Quality of Life in Essential Tremor Questionnaire (QUEST) is the only established syndrome-specific scale for quality of life of essential tremor [22] while generic scales like the Short Form (36) Health Survey [23] or the sickness impact profile [24] are occasionally used. For daily practice, the Archimedes spiral drawing together with the scoring of Bain [25] is a very useful follow-up.

The measurement and clinical weighting of treatment results is usually done by comparing the results of the abovementioned rating scales which grade tremor severity between 0 and 4. But the tremor amplitude has been shown to be logarithmically related to these clinical scores according to the Weber-Fechner law that perception is related logarithmically to the physical stimulus [26, 27].

The amplitude *T* (*T* = tremor amplitude, e.g., measured with a motion transducer) is logarithmically related to the tremor rating score (*R*) according to the formula:

1 $$ {\log}_{10}T=\propto R+\upbeta $$

Earlier studies have shown that α is between 0.4 and 0.6 for extremity tremors and β is typically between − 1 and − 3 [27, 28]. From this, the percent change of tremor amplitude can be calculated by:

2 $$ \mathrm{Percentage}\ \mathrm{change}=\frac{T\left(\mathrm{study}\ \mathrm{end}\right)-T\left(\mathrm{baseline}\right)}{T\left(\mathrm{baseline}\right)}=\left({10}^{\propto R\left(\mathrm{study}\ \mathrm{end}\right)-R\left(\mathrm{baseline}\right)}-1\ \right)\times 100 $$

Consider two patients with a hand tremor severity of 2 and 4 points on the TETRAS scale, (i.e., tremor amplitude of 1–3 cm or ≥ 20 cm, respectively. A reduction of 1 point for those would equal 50% and 25%, respectively, corresponding to a reduction of tremor amplitude from 1–3 cm to “barely visible” or > 20 cm to 5–10 cm. According to Formula 2, this would correspond to the same reduction of approximately 65% (assuming α = 0.5, β = − 2). With this approach, accelerometrically measured tremor amplitudes can also be used to determine the tremor reduction correctly. Similar to our previous work [29], we consistently use an α of 0.5 for the current study. All improvements of the tremor rating scales are reported here as percentage improvement according to this equation.

While the lack of a unified rating scale for tremors is a deficiency for all current rating, the reporting is further limited by inconsistent use of individualized subscores of the two main scales as outcome parameters in some studies. The different parts of the TRS (A, tremor exam; B, performance tests; C, daily activities) or the TETRAS (performance, tremor exam) are understandably separate outcomes, but sometimes outcomes for the upper extremities by combining items from the subscales or variable customized lateralized scores (e.g., combining physician rated tremor severity with performance tests) contralateral to the intervention are used. Others use single items like writing or just the postural tremor of the contralateral hand. There are even trials with outcomes differing between the original report [30] and the long-term follow-up study [31]. While such scores are needed to understand the value of the different interventions, unification of these outcomes will be needed to compare the results similar to other diseases like Parkinson’s disease or other better standardized fields of neurology. For the present evaluation, it adds to the heterogeneity of the results, despite the overall message remains robust. Our analysis has used lateralized scores, lateralized single items for action or postural tremor synonymously and separated from total scores if available.

## Selection of Studies

For studies using medication, the selection criteria of the MDS study group [32] were used and a PubMed search was done on January 3, 2020. For functional neurosurgical studies, only reports with ≥ 15 patients were selected. For head and voice tremor and for rare interventions, smaller subject numbers were accepted. Data from our earlier work was used [29].

## Treatment of Essential Tremor

### Non-pharmacological and Non-surgical Treatment of Tremor

Even simple interventions can improve the symptoms of mild to moderate tremor syndromes. Some medications (like certain antidepressants, antiepileptics, or inhalers) or foods (caffeine, energy drinks) can worsen tremor. Avoiding such drugs, if clinically possible, can improve tremor symptoms. Several, predominantly young patients report an increase in tremor after physical or muscular exertion. It is also known that periods of relaxation and sufficient sleep can improve the tremor symptoms [33, 34]. Given the emotional modulation of tremor relaxing techniques such as progressive muscle relaxation can be applied to improve tremor symptoms [35] .

Besides and in addition to pharmacological and invasive treatment options, a number of non-pharmacological therapeutic options are available. These include occupational therapy, speech therapy, and psychotherapy. Each of these individual therapies has its own specific role in the management of tremor.

Occupational therapy provides assessments to determine which tasks are particularly difficult for the patient and whether they are particularly accentuated in the area of extremities. Individualized therapy concepts can then be developed [36–38]. Occupational therapy provides skills that may help make everyday life functioning for individuals with tremor easier [39, 40]. The following supporting interventions have been proposed:

- Use of electric devices replacing mechanical handles (example: electric toothbrush)
- Use of weighted utensils (example: use heavy cup, weighted pens)
- Change dressing (example: elastic shoelaces)
- Use of electronic devices (example: speech-activated software, hands-free speakerphone features)

In vocal tremors, drug therapies often respond insufficiently [41–43]. For such patients, speech therapy may help [44, 45]. Treatment approaches pursue the following: (1) Relaxation and breathing exercises were to decrease tension in the head and neck, (2) facilitate increased airflow through the vocal folds during speaking to help achieve the targeted breathier and softer voice quality, (3) training of an easy voice onset to reduce effort during voicing onset and to promote increased airflow through the vocal folds, (4) use of a slightly elevated pitch during sentence repetition to discourage the down-ward pitch inflections [46].

Also social handicap, disability, mood disturbances, and anxiety are often part of a tremor syndrome. Face, head, and chin tremor are often perceived as stigmatizing. There is a high prevalence of embarrassment among individuals with ET leading to avoidance of social contacts and social withdrawal [47]. Psychotherapy helps to improve the individual’s well-being and mental health in tremor patients [48–50]. Psychotherapy includes various types of psychological therapies, the most widely used being cognitive behavioral therapy [51]. Although many tremor patients report to benefit from cognitive behavioral therapy, there are only few randomized studies available that show the impact of this accompanying treatment [15, 51–54]. Further trials in tremor syndromes are needed to shed more light on the effect of both individual and group cognitive behavioral therapy. These trials should include tremor patients in different stages of disease and should be designed to assess both motor and nonmotor symptoms. Thereby, the social environment and caring relatives should be involved into the treatment strategy.

### Medical Treatment of Essential Tremor

Historically, all drugs used for the treatment of essential tremor have been discovered by chance, when patients were treated with these drugs for other reasons and tremor improvement was observed as a side effect [55, 56]. Just recently, the first drugs are developed addressing mechanisms which are likely to interfere with central mechanisms of tremor such as selective voltage-activated calcium channel antagonists that show low nanomolar potency against all 3 Cav3 isoforms which are involved in oscillatory properties of neurons [57] or octanoic acid relying on the tremor-reducing effect of alcohol [58] without the dependency developing effect [59]. But efficacy is not yet fully established and it will take time until the first drugs based influencing the underlying mechanisms will be available. Regarding the currently used drugs, our understanding of their effects on tremor is sparse. Just globally, widespread pathological oscillations occur within the motor system in patients with ET, and drugs presumably interfere with these oscillations [60].

The pharmacological treatment has been reviewed in the past decade [29, 61, 62]. The recent evidence-based review of the Movement Disorder Society has defined the latest standard [32].

The two most established drugs propranolol and primidone were tested in the 1970s and 1980s of the last century with small numbers of patients and for periods of less than 3 weeks and trial designs which do not match current standards of assessment while topiramate was more recently studied. The first clinical scale, the Fahn/Tolosa/Marin scale (TRS) is available only since 1993 [18] and was validated only in 2012 [19]. Most of the older studies used accelerometric measurements which have a log relation to clinical scales for reasons mentioned above. As this was only discovered late [27, 28], the investigators left all accelerometric measurements in the early 1990s and their use is just nowadays becoming more popular with the emergence of wearable motion sensors [63].

### Propranolol

**Fig. 1:**
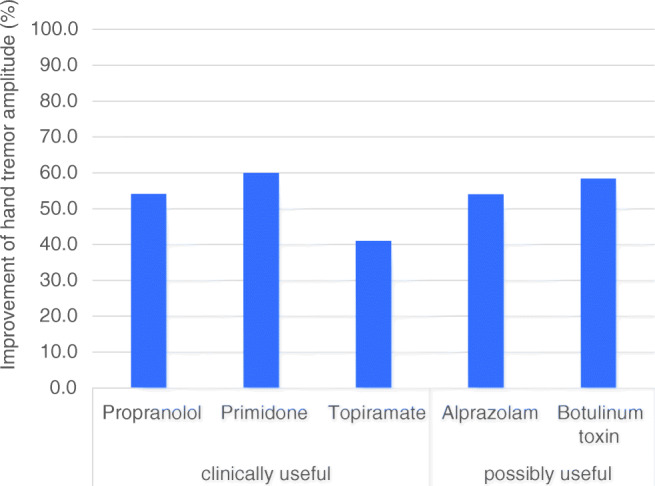
Drug interventions for essential tremor. The mean improvement of the tremor scores are shown (study selection see text). The suppression of tremor amplitude is shown for the different drugs. They are grouped according to MDS criteria of clinical usefulness [29].

Among the drugs of first choice for ET is propranolol [32, 64], a nonselective β-adrenergic receptor antagonist. The effect is likely exerted on central β2 receptors [65] but peripheral effects on the muscle spindle are also present [66]. As propranolol also has major cardiovascular effects, an electrocardiogram should be carried out before starting the treatment. Propranolol is contradicted in cases of severe bradycardia or second- and third-degree AV block. Generally, propranolol is well tolerated at lower dosages but side effects include hypotension, fatigue, depression, and erectile dysfunction [67]. It is contraindicated for patients with asthma and should be used with caution in diabetes patients because of its masking effect on hypoglycemia. Daily doses of 30–320 mg (mostly 60–240 mg) are recommended (Table 4) [43]. Low daily dosages (30–60 mg) can be tried for small amplitude tremors. Intermittent treatment in stress situation is used by some patients. Some artists regularly use this to improve their performance. Short-term improvements are in the order of 30–75% (average 44%) and mainly based on accelerometric measurements (Fig. 1) [29]. Long-term experience is limited and based on uncontrolled case series. One-third of the patients or more had no benefit, chronic side effects were found in 17%, and 17% developed tolerance to propranolol [68, 69]. As a rule of thumb, half of the patients have a long-term benefit from propranolol and have a 50% reduction of tremor severity. There are no known predictors for a positive response. It is approved for tremor treatment in most countries.

**Table 4:** First-line drugs for the treatment of ET

Drug	Dosage
Propranolol	30–240 mg/day
Primidone	< 30–500 mg mg/day
Topiramate	400–800 mg mg/day

### Primidone

Primidone is an anticonvulsant supposed to enhance GABAergic inhibition [70]. Antitremorogenic effects of primidone can be observed at plasma levels that are lower than those used for treatment of epilepsy [71]. Recommended dosages are from 30 to 500 mg/day (Table 4) [72, 73]. Primidone often causes acute side effects with vertigo, nausea, dizziness, and fatigue, which can be reduced with very low dosages at the start of treatment [72]. Drowsiness, dizziness, and rarely depression and cognitive and behavioral effects are long-term side effects. Again, the quality of studies documenting the effect is low. The mean effect of tremor reduction which is based on six studies is 60% (Fig. 1) [29]. Again, approximately 50% have a response to treatment with occasional patients with a dramatic response. Predictors of the treatment effect are lacking. Unfortunately, the drug is not approved for tremor treatment in most countries.

A further therapeutic option is the combination of propranolol and primidone at the maximum tolerated dose [74].

### Topiramate

Topiramate has been upgraded as a first-line treatment [32] based on a better study quality for this drug compared to propranolol and primidone despite some heterogeneity of the results [75]. It is an antiepileptic and the reason for its specific action on tremor is unidentified, but it is enhancing the GABAergic transmission and the blockade of voltage-dependent calcium and sodium channels may also play a role [76]. Four studies [77–80] were placebo-controlled and had an overall of 314 participants. Two meta-analyses [75, 81] are available. Frequently observed side effects are as follows: weight loss, paresthesias, trouble concentrating, and memory disturbance, and an increased risk of kidney stones. It is contraindicated in pregnant women or women with child-bearing potential without contraception. The mean improvement is 41% (Fig. 1). Dosage is critical and higher dosages of 200 mg are most likely needed, because the only negative study had lower dosages (Table 4) [77]. In most countries, topiramate is not approved for the treatment of tremors (Table 4).

### Botulinum toxin

Botulinum toxin injected in wrist flexor and extensor muscle has been tested in two double-blind 16-week studies [82, 83]. Staged injections between 50 and 100 μg were used. The effect is estimated between 50 and 68% when accelerometric or clinical data were used. Improvement was accompanied by weakness in 30% of the low-dose and 70% of the high dose group with a severity of up to 30% of the baseline grip strength. Two further studies with significant improvements were meanwhile reported [84–86]. Functional improvement measured with the sickness impact profile was not significant. It is a treatment which needs highly specialized skills of the physician and extensive experience. Additionally, electromyography or ultrasound imaging can improve the result although one study questioned the value of ultrasound imaging or EMG for the improvement of treatment results [86]. Despite only small studies, treatment of voice tremor with botulinum therapy is established [42, 87–89] and considered the standard for severe voice tremor. Given the risks of severe side effects such as severe breathiness and difficulty swallowing associated with botulinum toxin injections, experience is needed and a close collaboration with laryngologists (Fig. 1).

## Further Drug Treatments

Other beta-blockers have been found to be useful, although in small studies. Atenolol was used in three studies [90–92]; sotalol was used in two studies [90, 92]. Metoprolol as a β1-selective blocker has been studied to understand the locus of action of β-blockers for tremor, but unfortunately the potency of metoprolol as an antitremor drug [92–96] is not adequately studied. Despite the very common use of metoprolol for cardiologic indications, this important clinical question cannot be adequately answered. Alprazolam was tested in 2 studies [97, 98]. For gabapentin, one study showed no effect [99] and two studies found a 50% improvement [100, 101].

Clonazepam has been reported to be useful in ET with intention tremor [102] but another small study could not reproduce this [102]. Clozapine has been reported to be effective if a test dose (6.25 mg) shows a positive effect [103]. Clozapine is administered at night for its sleep-inducing effect. It is not approved for essential tremor and clozapine can cause leukopenia, particularly for neutrophil leukocytes in 5% of the patients, rarely a life-threatening agranulocytosis and in some cases additional thrombocytopenia. Frequent blood count checks must be carried out, more often in the first weeks and months but as long as treatment is maintained. Other possibly efficacious drugs have been reported like flunarizine [104], which also has the potential for parkinsonism as a side effect. Theophylline has been shown in a small double-blind study to be effective [105]. All these drugs are only rarely used in daily practice.

For some drugs, lack of efficacy or no significant benefit has been demonstrated like levetiracetam, trazodone, pindolol, acetazolamide, mirtazapine, 4-amino-pyridine, mirtazapine, pregabaline, nifedipine, and verapamil [32].

### Invasive Treatments for Essential Tremor

The treatment of tremors with surgical interventions has a long history dating back to the 1950s of the last century. Initially based on the observation of spontaneous accidental strategic lesions, a specific region of the ventral thalamus, the nucleus ventrointermedius (Vim), and the region below this nucleus in the so-called zona incerta [106] was identified as the best place for the intervention [107, 108]. This is the location where destruction of cells and fiber tracts with radiofrequency heating in the last century and just recently with focused ultrasound can improve tremors. This is also the location where the electrodes of deep brain stimulation are placed and the presumed mechanism for this beneficial effect is the activation of the glutaminergic cerebello-thalamic and cortico-thalamic projections at high frequency leading then to a synaptic fatigue of these terminals and thus the pathologic rhythmic activity is blocked [109]. The mechanisms underlying tremors are complex and they likely differ between the different tremor syndromes. However, converging data from electroencephalographic [110], magnetoencephalographic [111] recording and functional MRI imaging [112] suggests that they finally feed into a tremor network consisting of a cerebello-thalamo-cortico-cerebellar loop. One major relay station, a bottle neck, of this tremor network is the thalamic Vim which makes the therapeutic effect with lesioning or electrical blockade of this core region quite understandable.

Vim-DBS, Vim lesions with radiofrequency heating, or focused ultrasound and radiosurgery are available for tremor treatment. By far the largest group of patients are patients with essential tremor but other tremor disorders have been treated as well. Radiofrequency lesioning of the Vim has been the mainstay of functional tremor surgery for decades but this has almost been abandoned when DBS became available in the 1990s in many countries due to the adaptability of DBS. Radiosurgery is a technique practiced only in a handful of centers worldwide. The MR-guided focused ultrasound (MRgFUS) is a new lesional technique which is increasingly used. Each of these techniques has its pros and cons.

### Radiofrequency Lesioning of the Vim

Radiofrequency lesioning is a functional neurosurgical technique performed with a special electrode through which high-frequency current produces a local heating of the tissue at the tip of the electrode above 60 degrees leading to the destruction of all cells and fiber tracts in the target region. The extent of the lesion is determined by the strength and duration of the electrical current. Afterwards, the heating electrode is removed. The patient’s head must be rigidly connected to a frame around the head and imaging of head and frame on CT or MRI allows defining the target coordinates where the electrode is then placed with high-precision instruments. The planning of the electrode tract is performed on the basis of this head/brain/frame image. There are standard coordinates where the tip of the electrode was placed in the past. The VIM is indirectly targeted on the MRI scan. Nowadays, the individual brain structure on MRI imaging (anatomical position of anterior commissure, posterior commissure, and the third ventricle) can be used to further guide the lesion location. Temporarily used microelectrodes allow locating the best target based on cell recordings or the suppression of tremor in the awake patients while stimulating with electrical high-frequency pulses through this microelectrode.

**Fig. 2:**
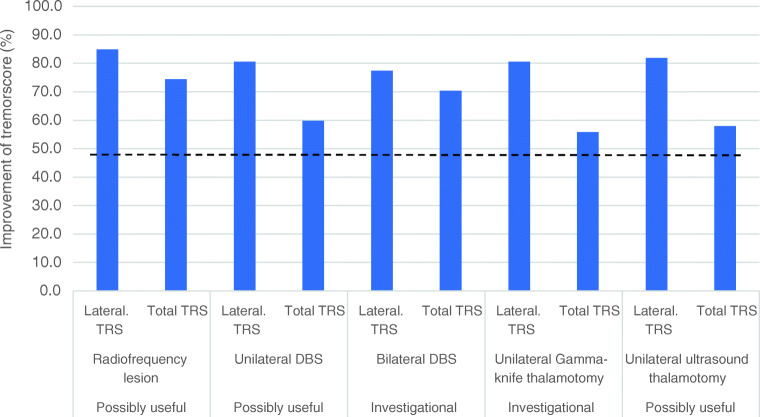
Surgical interventions for essential tremor. The four options are shown with the grading of usefulness of the Movement Disorder Society [29]. The bars show the mean improvement in percent for the different interventions reported in the studies mentioned in the text. The lateralized TRS is summarizing tremor severity items only on the side contralateral to the intervention. The total TRS covers tremor severity, performance tests, and activities of daily living. The dotted line is the average improvement of the lateralized score exerted by medications (see Fig. 2). The grading of clinical usefulness does not reflect the strength of the effect size of the intervention but the strength of published evidence.

Thousands of patients have been treated with radiofrequency lesioning in the last century [106, 113] [114]. The reports are not compatible with current standards for trials although efficacy transpires from their reports. Few modern studies are available which use standardized assessment [115, 116]. The improvement of the lateralized outcomes is 84% and for the tremor total score around 74% with very few data [115–117]. The most important limitation is the irreversibility of the procedure and the higher adverse event rate. Main surgical side effects have been reported to be more common for radiofrequency thalamotomy than for DBS [117, 118]. A meta-analysis including 225 patients found speech disorders in 4.5% after unilateral and in 13.9% after bilateral lesioning [119]—more frequent after left- than after right-sided lesions (Fig. 2). Thus, radiofrequency lesioning is only applied unilaterally and allows the creation of small and localized lesions. Its effectiveness and low cost made this procedure suitable for the surgical treatment of tremors in the past [120–122] but due to the high incidence of side effects, it has almost been abandoned

Long-term effects are controversial and only poorly documented [113] but it is likely that for ET, there long-term efficacy is similar as for the other better studied procedures [123, 124].

### Deep Brain Stimulation for Tremors

Deep brain stimulation is a functional neurosurgical technique by which a permanent electrode with 4–8 contacts is implanted to the Vim of the thalamus with stereotactic planning of the target with or without confirming the target area with microelectrodes in the awake patient. The stimulator is implanted subcutaneously in the subclavicular area and a subcutaneously implanted wire connects to the electrode. The current applied through the contacts of the permanent electrode is blocking cells and fibers in the target area and the radius of this functional and reversible blockade is depending on the strength and pulse characteristics. It is currently the standard of surgical interventions for tremor.

There are many prospective, open studies and case series case observations, but a randomized trial of Vim-DBS against best medical treatment is still lacking. The current evidence has been reviewed recently [29, 125–128]. The qualitatively best study is a prospective, uncontrolled multicenter trial (*n* = 126 patients) stimulated unilaterally (*n* = 79) or bilaterally (*n* = 47) with video-blinded outcome of 76 patients [129]. The best target for stimulation is a matter of controversy over decades if the Vim itself or the posterior subthalamic area below the Vim. This has been discussed for radiofrequency lesioning [106, 130] and particularly for DBS. DBS studies have shown that the effects for the PSA may or may not be superior [131–134] but the PSA can be stimulated with lower currents [135]. Most studies are using the Fahn tremor scale [136] (TRS) as the main outcome parameter. The mean improvement of DBS (cumulated data from 14 studies [129, 137–147]) for the contralateral tremor is 87% and the total tremor score is 66%. The largest trial has shown a significant improvement of the lateralized score improved by 85% at 6 months and the total score improved by 75%, assessed by blinded reviewers [129]. Later publications are also using a quality of life score (QUEST) [49] and the improvement of unilateral Vim-DBS was significant [148] and reported in one study to be more than 70% after bilateral stimulation (Fig. 2) [149].

Axial tremors (head and voice tremor) are known to be less responsive to medical but also surgical treatments [29] than extremity tremors. Particularly, unilateral DBS was found less efficient than bilateral DBS in several studies for head and voice tremor [29] which has led to the recommendation of bilateral procedures in case of axial tremors. A secondary analysis of the so far largest study on uni- and bilateral DBS [129] has found that unilateral stimulation has not only also some ipsilateral effect on hand tremor but also a significant effect on midline tremors [150]. Bilateral stimulation further improves the midline tremors [150] suggesting a staged procedure for head tremor to be justified.

In a recent prospective study [129], quality of life measured with the QUEST improved by 33% for the first year. One retrospective study with patients undergoing bilateral DBS or FUS treatment had a similar improvement of quality of life [149]. Most reports show an improvement of quality of life during the first years but beyond 5 years this effect seems to disappear [151].

The long-term tremor suppression of patients with ET is currently debated. There are patients developing short-term habituation with a reduction of effect within hours or days. This may be due to a relative misplacement of the electrode or other reasons which can be treated [152]. Despite there is still a significant effect of Vim-DBS after 10 years or beyond if the tremor suppression between ON and OFF stimulation is compared [137, 139], there seems to be a tendency for a loss of effect when compared with the baseline which is also recognized by the patients [153]. It is unlikely that this is only due to disease progression [138] but this needs further investigation [154].

Much is known about the side effects of DBS and particularly for Vim-DBS in ET. A recent review [155] found the average rates for intracranial complications to be 3.4% for pooled analysis of patients from different studies. The annual hardware removal rates were 2.4% for per-patient analysis and lead revision rates were 2.6%. A recent report two US databases for interventions found much higher rates of surgical revisions or removals [156]. The reasons for this are not well understood and may have to do with the widespread application versus specialized centers for DBS. Surgical side effects are similar for most indications but differ for syndromes in quantitative terms. For example bleedings and stroke occur more often in PD patients (2%) than in ET patients (0.74%). Among the risk factors for bleedings are possibly the number of microelectrode tracks used to find the best target but this is still controversial [155, 157]. An increased risk likely exists for hypertensive patients [158]. Another common problem are infections which occur in 3–10% depending on the study [159–161]. Infection rates vary widely in the literature. Among DBS-related infections, skin infection and hardware-related complications are the most common ones. Intracranial infections need explantation of the system which is not necessary for infections along the cable or pulse generator [159–161]. DBS surgery has on average a morbidity rate of 3.7%, which is mainly caused by intracranial hemorrhage [162, 163]. The use of highly developed treatment planning software together with multiplanar three-dimensional imaging or the restricted application of intraoperative electrophysiology lowers the morbidity considerably [162]. To compare efficiencies, identify workflow obstacles, and reduce the morbidity rate, main steps in DBS surgery workflow had been described as standard operating procedure [164]. Stimulation can cause also adverse events. Adverse events like muscle contractions, paresthesia, dysarthria, and limb or gait ataxia can occur in most patients with increasing stimulation strength depending on the lead location and are usually explored during the monopolar review of the therapeutic window of each single contact early after surgery. The aim is to adapt stimulation strength to an amount which gives a satisfactory result of tremor suppression without such side effects. This is usually possible for persistent paresthesias. For ataxia and gait problems, this may be difficult and is not infrequent in clinical practice. It has been found that this may be due to stimulation of retrograde fibers to the cerebellum. Following this study, it is a reversible stimulation-induced side effect [165]. Also speech disturbances are common and have been found in a meta-analysis to be as common as 12.3% after unilateral and 41.4% after bilateral Vim surgery [119]. Again adaptation of the stimulation strength can help but sometimes speech disturbances occur already at stimulation strengths which just provide sufficient tremor reduction. For long-term treatment, this can be one of the problems which cannot be properly solved. Nevertheless, Vim-DBS represents a highly effective and safe treatment method which is the current standard, even though it is associated with high costs.

### Radiosurgery for ET

Radiosurgery is done in the radiation suite and uses focal radiation to destroy the tissue in the target area, again in the well-defined Vim through imaging of the rigidly fixed head. There is no reliable way to test the individual response in a patient as for the other methods.

The use of radiosurgery is rare and limited to few highly specialized centers worldwide without a clear tendency for growth but reports date back to the 1990s [166]. A special feature of this treatment is that effects and also side effects can only be seen after weeks or months. The delayed effect is due to post-radiation reactions as subacute tissue reaction such as scarring following. A first blinded evaluation of 14 patients did not find a significant improvement of tremor items except for a small improvement of the TRS, part C. However, three subsequent studies found an improvement of the total TRS of 55.9% and of 81% for tremor [167–169]. Long-term effects of 17 out of 52 patients which were followed up to 4 years were reported to be stable [170]. Side effects are reported to be as rare as 0.7% in a recent meta-analysis. At least they are highly variable and cases with running lesions and further complications do not show up in the reports. They are reported for single cases [171, 172]. Radiosurgery is carried out unilaterally in the majority of cases. The surgery is inexpensive compared to other methods. Due to the small number of interventions carried out, there is no reliable data on the long-term effect of the intervention for periods longer than 4 years.

### Focused Ultrasound

MR-guided focused ultrasound is using 1024 synchronized ultrasound emitters which are focused to a single point. When the temperature increases above 50°C, the protein is denatured and the cells and fibers are irreversibly destroyed. The head, again rigidly fixed in a stereotactic frame, is placed exactly with the target area into this focus. Identifying the target area is done analogously as with the abovementioned techniques. Confirmation of the target with microelectrodes is not possible due to the incisionless technique, but this can be replaced by heating the target region to ~ 48°C which reversibly inactivates the tissue and thereby the effect on tremor can be tested clinically before a definite lesion is placed with higher temperature. The head has to be carefully shaved for this procedure.

Despite focused ultrasound has been introduced only few years ago, the quality of evidence for the treatment is the best because there is a randomized trial compared to sham treatment [30]. So far there is reliable data only for unilateral stimulation. This showed a 71% improvement of the lateralized score compared to 3% in the sham group and a 41% improvement of the total tremor score compared to 2% in the sham group. The improvements for the four open-label studies [149, 173–175] were similar and not unexpectedly even better (Fig. 2). Most studies are using the Vim as the target but as for DBS or radiofrequency lesioning, some groups are using the entry region of the cerebellothalamic tract [176, 177], first described by Velasco [107]. The results are very similar for both targets.

Effects at 2 years [31, 178] and up to 4 years [179] have been reported. Overall, for this period, the results were stable with a possible small decline. No additional adverse events occurred [180].

Scores for head and voice tremor improvements are not specifically shown, but some studies mention no [181] or only mild [30, 175] improvement of these midline tremors.

A pooled analysis of the complications of therapy of 170 patients reported rarely severe side effects (1.7%). Paresthesia, numbness, ataxia, and disturbances of balance are persistent for 12 months in 18% of the patients; however, the severity is mostly mild. Bleedings and infections did not occur.

Importantly, with the currently available technology, patients are only eligible if the skull density ratio, a measure of the penetrability of the skull for ultrasound, is above 0.35–0.4 [182]. This measure can be calculated from routine CT scans of the head. Compared to deep brain stimulation, focused ultrasound is less expensive [183, 184].

### Comments on the Pragmatic Treatment of Essential Tremor

Patients with essential tremor seeking advice from the neurologist want to gain insight into the origin of their tremor and/or want to obtain treatment. Therefore, the first step is to educate the patients about the syndrome and treatment options. Given the fact that ET is a chronic syndrome with only symptomatic therapies, the search for non-medical and non-surgical therapies is important. Indeed, a large portion of patients is coping with the condition without any medical therapy. Of those who seek treatment, one-third stops medication [185]. We estimate that less than half of the patients with ET ever try treatment. This is not only the patient’s decision, as still nowadays, many general physicians consider monosymptomatic tremor as a fate rather than a disease. Medical and surgical interventions are therefore not regularly offered.

**Fig. 3:**
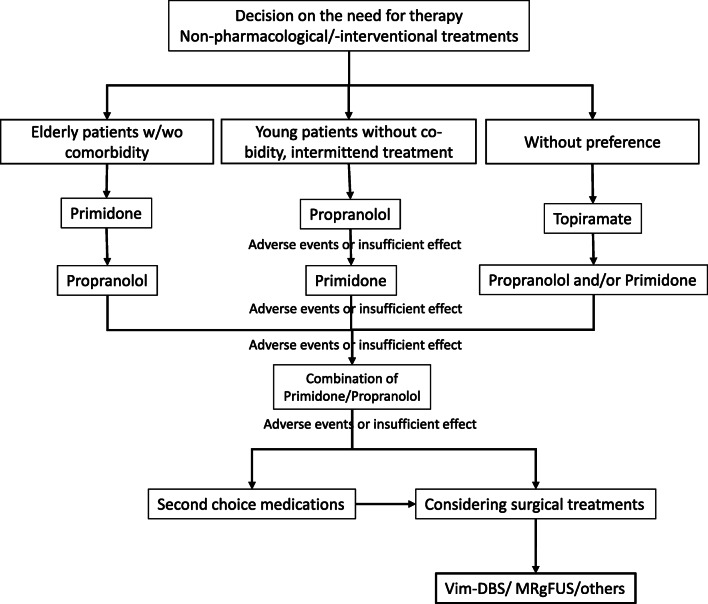
Decision tree for the treatment of patients with essential tremor.

If the outcome of the discussion with the patient is for treatment, the optimal therapy has to be chosen. Figure 3 summarizes the treatment approach in our center. Usually the first step is drug treatment. The three main drugs for tremor all have a similar efficacy despite some doubts on the quality of evidence. The drug of choice for a particular patient is therefore mainly driven by side effects. For younger patients and intermittent treatment, propranolol may have advantages while primidone may be easier tolerated by elderly people. Topiramate should not be used in patients with nephrolithiasis, pregnant women, or women with child-bearing potential without contraception. The weight reducing potency is a concern for some patients and a wanted side effect for others [186, 187]. Trying other than these three drugs is mostly not promising and the patients’ preference may decide on continuing the testing of further drugs. Approximately 30% stop taking medication after one or two failed and even after successful attempts [185] and lack of success may demotivate patients. On the other hand, the discussion of advanced treatments should not be prolonged. It is obvious from the data reported above that the surgical interventions are much more powerful than medication and for severe tremors they are the only treatments which can provide sufficient symptom control.

The severity of tremor and its subjective perception by the patient and the specialist/patient interaction decides on choosing invasive treatments or not. The large portfolio of interventions has grown in the past years. But the discussion about invasive therapies will naturally focus on the available possibilities in a given healthcare system. Radiosurgery is available only in very few countries. Focused ultrasound is also still only rarely available although rapidly growing. Most countries do have DBS teams which is currently the standard of care in many countries. The advantages are the vast experience, the adaptability of stimulation strength, and the well-known and mostly manageable side effects and with good tremor control for many patients. The current common sense is that patients with severe bilateral tremors or patients with midline tremors as the target symptom need bilateral procedures. For them, DBS is the standard option. But it is meanwhile well documented that unilateral procedures may also have significant and likely clinically meaningful effect on midline tremors. Staged procedures with treatment for the dominant side first are common in many places and may be attractive for such patients but have also the disadvantage of two interventions. The side effects of bilateral lesioning are unacceptable for lesional radiofrequency and unknown for radiosurgery. For FUS, it is not explored and it is unclear if bilateral procedures would be better tolerated than for radiofrequency surgery (Table 5).

**Table 5:** Aspects which might be considered for the treatment choice during the shared decision-making between patient and physician

	Deep brain stimulation	Focused ultrasound thalamotomy	Gamma-knife thalamotomy	Radiofrequency thalamotomy
Availability	Many centers worldwide	Few centers to-date but rapidly increasing	Extremely rare	Rare
Experience	Vast experience	Limited experience	Only few centers offer this treatment	Only few centers offer this treatment
Bilateral treatment necessary	Established option	Only within clinical studies	Almost no data	Not recommended because of side effects
Exclusion criteria due to patient’s risk profile	Multimorbidity as for other brain surgeries, frailty	Frailty often tolerated	unknown	Multimorbidity as for other brain surgeries, age (?)
Patients perception of procedural risks	Open brain surgery	Incision-free but invasive	Incision-free but invasive	Open brain surgery
Procedure under general anesthesia possible	For rare cases, but carries special risks	Not established	Not established	Not established
Risk of infections/bleedings	Possible, but rare	Fewer than DBS, limited experience	No reported with limited experience	More frequent than for DBS
Need to shave the head	partial	Complete	no	partial
Single-stage treatment	Yes, can be done within one surgery or staggered	Yes	Yes	Yes
Exclusion because of skull density	no	20-30% of the patients	no	no
Experienced center for follow-up needed	needed	Not regularly needed	Not regularly needed	Not regularly needed

The diagnosis is key for the selection of the target for intervention and one of the important differential diagnosis of essential tremor is dystonic tremor. The boarder between these two conditions is not very well defined. Tremors in the setting of indisputable dystonia are usually treated with interventions in the Gpi based on the lesson from published data on tremor improvement in focal, segmental, and generalized dystonia [188]. On the other hand, essential tremor patients need Vim surgery. There is still uncertainty about the target for patients with the presenting symptom of tremor who have soft signs for dystonia. While several reports have shown good results for such patients with Vim surgery [139, 189, 190], there are patients who have no or no sufficient improvement. There is no scientific solution to this problem and most centers target the Vim and the neighboring zona incerta for these cases. DBS has the advantage that one contact of the electrode can be placed in the gray substance of the Vim and another just below in the zona incerta of which the radiation prelemniscalis is part.

The final decision for or against a specific intervention depends on many factors which do not necessarily apply for all patients but should be discussed with the patient. Table 3 summarizes aspects which we usually cover during shared decision-making between patient and physician, and the information we offer is naturally not always based on proven evidence. Some aspects like availability, experience with the method, choice for bilateral treatment, and exclusion because of multimorbidity are more or less defined by the circumstances other factors are specific for one or several of these interventions. A detailed explanation of the surgical risks is important. For focused ultrasound, the complete shave of the head is concerning for some. Some patients cannot receive the treatment because their skull is too dense. Focused ultrasound is an invasive treatment, but given the relatively short-term procedure in the MRI, it can also be offered to elderly patients which otherwise would not qualify for surgical interventions. This applies in principle also for radiosurgery. Patients living remote from an experienced team sometimes prefer interventions which do not need regular follow-up visits. For most patients, the expert experience of the center is key.

Treatment options for essential tremor are much better than frequently thought. The burden of disease for essential tremor is high. Based on the current estimation of prevalence [191], 69 Mio patients worldwide have the diagnosis of essential tremor [192]. An unknown percentage of these patients need therapy. Neurologists are the natural advocates of ET patients which should point out the customized possibilities for treatment and allow patients’ decisions on the needs. In the future, the combination of validated scales with new sensor-based measurement tools will most likely improve the quality of future studies.

## Electronic Supplementary Material

ESM 1 (PDF 1224 kb)
