# Correction to: Managing Essential Tremor

**DOI:** 10.1007/s13311-021-01131-5

**Published:** 2021-10-25

**Authors:** Franziska Hopfner, Günther Deuschl

**Affiliations:** 1 Department of Neurology, UKSH, Christian-Albrechts-University Kiel, Rosalind-Fraenklinstr. 10, 24105 Kiel, Germany; 2 Department of Neurology, Hannover Medical School, Hannover, Germany

## Correction to: Neurotherapeutics volume 17, pages 1603–1621 (2020) 10.1007/s13311-020-00899-2

The article “Managing Essential Tremor,” written by Franziska Hopfner and Günther Deuschl, was originally published Online First without Open Access. After publication in volume 17, issue 4, page 1603–1621, the author decided to opt for Open Choice and to make the article an Open Access publication. Therefore, the copyright of the article has been changed to © The Author(s) 2020 and the article is forthwith distributed under the terms of the Creative Commons Attribution 4.0 International License, which permits use, sharing, adaptation, distribution, and reproduction in any medium or format, as long as you give appropriate credit to the original author(s) and the source, provide a link to the Creative Commons license, and indicate if changes were made. The images or other third party material in this article are included in the article’s Creative Commons license, unless indicated otherwise in a credit line to the material. If material is not included in the article’s Creative Commons license and your intended use is not permitted by statutory regulation or exceeds the permitted use, you will need to obtain permission directly from the copyright holder. To view a copy of this license, visit http://creativecommons.org/licenses/by/4.0/.

